# Advances in Bioceramics for Bone Regeneration: A Narrative Review

**DOI:** 10.3390/biomimetics9110690

**Published:** 2024-11-12

**Authors:** Baylee M. Brochu, Savanah R. Sturm, Joao Arthur Kawase De Queiroz Goncalves, Nicholas A. Mirsky, Adriana I. Sandino, Kayaan Zubin Panthaki, Karl Zubin Panthaki, Vasudev Vivekanand Nayak, Sylvia Daunert, Lukasz Witek, Paulo G. Coelho

**Affiliations:** 1 University of Miami Miller School of Medicine, Miami, FL 33136, USA; 2 Department of Biochemistry and Molecular Biology, University of Miami Miller School of Medicine, Miami, FL 33136, USA; 3 Biomaterials Division, NYU Dentistry, 345 E. 24th St., Room 806, New York, NY 10010, USA; 4 Department of Biomedical Engineering, NYU Tandon School of Engineering, Brooklyn, NY 11201, USA; 5 Hansjörg Wyss Department of Plastic Surgery, NYU Grossman School of Medicine, New York, NY 10016, USA; 6 Division of Plastic Surgery, DeWitt Daughtry Family Department of Surgery, University of Miami Miller School of Medicine, Miami, FL 33136, USA

**Keywords:** bone tissue regeneration, bioceramics, bone grafts

## Abstract

Large osseous defects resulting from trauma, tumor resection, or fracture render the inherent ability of the body to repair inadequate and necessitate the use of bone grafts to facilitate the recovery of both form and function of the bony defect sites. In the United States alone, a large number of bone graft procedures are performed yearly, making it an essential area of investigation and research. Synthetic grafts represent a potential alterative to autografts due to their patient-specific customizability, but currently lack widespread acceptance in the clinical space. Early in their development, non-autologous bone grafts composed of metals such as stainless steel and titanium alloys were favorable due to their biocompatibility, resistance to corrosion, mechanical strength, and durability. However, since their inception, bioceramics have also evolved as viable alternatives. This review aims to present an overview of the fundamental prerequisites for tissue engineering devices using bioceramics as well as to provide a comprehensive account of their historical usage and significant advancements over time. This review includes a summary of commonly used manufacturing techniques and an evaluation of their use as drug carriers and bioactive coatings—for therapeutic ion/drug release, and potential avenues to further enhance hard tissue regeneration.

## 1. Introduction

Bone is a complex, metabolically active tissue that provides both structural stability and dynamic adaptability for regeneration [1]. It is a composite material, primarily comprised of a network of collagen fibers reinforced by hydroxyapatite (HAP), a mineral phase of calcium phosphate (CaP) crystals [2]. Despite the individual fragility of collagen and HAP, bone exhibits remarkable structural strength due to its architecture. At the microscopic level, HAP crystals are oriented parallel to collagen fibers and arranged periodically, thus giving bone its lightweight yet mechanically robust properties [3]. Additionally, collagen facilitates bone reactivity by serving as a reservoir for different ions, notably calcium and phosphate, which are crucial for bone mineralization—the ability of bone to dynamically adapt in response to variations in load demands [4]. Furthermore, bone mineralization is mediated by osteoclasts and osteoblasts in response to various stimuli such as decreased serum calcium or phosphate levels [5].

Bone regeneration is a complex process driven by the interplay of cellular activity, biochemical signals, and mechanical stress. Osteogenesis, a continuous physiological process, is activated during injury. Inflammation initiates the foundational process of bone repair, which involves the recruitment, proliferation, and differentiation of mesenchymal cells. However, these processes depend on a supportive physiological environment [6]. Critical factors include stabilization of the injury site through granulation and cartilaginous tissue formation, degradation of damaged tissue by inflammatory cells, and angiogenesis. Conditions at the fracture site—including hormones, nutrients, pH, mechanical stability, and electrical environment, along with the roles of periosteum and bone marrow—have been indicated to be critical to the healing process [6]. While not all these factors are fully understood, current research and technology aim to leverage this knowledge to develop devices that enhance bone repair.

Bone’s intrinsic regenerative capacity is limited to “small” bone defects. Larger defects, such as those resulting from trauma, tumor resection, or fracture nonunion, exceed native healing capacity and require a bone graft to ensure adequate healing and enable the restoration of form and function at the defect [2]. In the United States alone, over 2 million bone graft procedures are performed each year, rendering this an important area of investigation and research [7,8]. An ideal bone graft is described to be structurally robust, osteoconductive, osteoinductive, osteogenic, cost-effective, and associated with minimal complications [5]. To date autografts remain the gold standard choice. Since they come directly from the patient and are native bone, they are naturally osteogenic and have a low risk of disease transmission [9,10]. Autografts are sourced either from cancellous bone, most commonly from the iliac crest, or from cortical bone. Cancellous bone contains osteocytes, stem cells, and growth factors making it highly osteoinductive and osteoconductive. While it initially does not provide much structural support, mechanical stability is rendered to the grafted site over time as bone regeneration progresses at the defect site. Cortical bone, on the other hand is less biologically active, relative to cancellous bone, and takes longer to revascularize. This is offset by increased structural support, and is used more frequently to span various types of large bone defects [11]. However, autografts are associated with significant disadvantages including donor site morbidity, extended recovery time, and the need for a secondary surgical site [12]. Donor site morbidity is one of the greatest concerns pertaining to the use of autografts. This was previously demonstrated by a retrospective review of 243 autogenous bone grafts by Younger et al., where a major complication rate of 8.6% (including infection, prolonged wound drainage, reoperation, pain persisting more than 6 months, and sensory loss) was observed, coupled with a higher minor complication rate (20.6%) [13].

**Figure 1:**
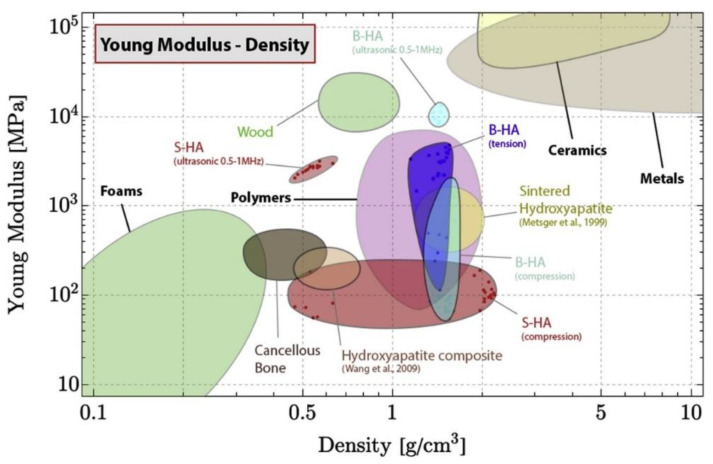
Ashby chart showing the mechanical behavior of various materials relative to apatite and bone (B-HA = apatite, S-HA = sintered HAP), Reprinted with permission from Ref. [17], Copyright 2020, Elsevier. The Ashby chart highlights how synthetic graft materials may or may not closely mimic the mechanical properties of native bone—a parameter crucial for preventing complications such as stress-shielding. The graph serves as a valuable tool to identify and evaluate novel materials with suitable mechanical properties for bone graft applications. Additionally, it suggests the potential for combining materials to render unique properties that optimize both mechanical performance and biological integration. By mapping how different materials perform under stress, the figure facilitates selection of materials for bone grafting.

Due to these limitations, allografts, xenografts, and alloplasts (e.g., synthetic bone grafts) are being explored as viable alternatives. Allografts and xenografts can be procured in greater quantities relative to autografts; however, they present increased risk of potential disease transmission and immune-mediated rejection [9,14]. Synthetic grafts present as a potential alterative due to their patient-specific customizability, but presently lack widespread acceptance in the clinical space [2]. Early in their development, non-autologous bone grafts were synthesized utilizing metals such as stainless steel and titanium alloys due to their biocompatibility, resistance to erosion, mechanical strength, and durability. However, risk of failure over time due to their differences in mechanical properties compared to native bone, commonly termed stress-shielding, was noted as a primary concern [15]. The possibility of tissue dehiscence, the necessity for a second surgical procedure, relatively high immunogenicity, and lack of angiogenic capacity rendered metals as less-than-ideal bone graft substitutes, leading engineers and researchers to explore novel materials with comparable physicochemical characteristics to that of native bone (Figure 1) [16].

Apart from the commonly utilized metallic-based tissue engineering devices, the field of biomaterials has also incorporated bioceramic- and polymeric-based materials [18,19]. Polymers are materials composed of long chains of atoms joined by natural or synthetic covalent bonds and are used with or without the addition of cellular components or biological mediators [18,20]. Most scaffolds studied for bone regeneration include natural polymers, such as chitosan, hyaluronic acid (HyA), and collagen (COL), as well as synthetic polymers such as polylactic acid (PLA) and polycaprolactone (PCL) [21,22,23,24,25]. On the other hand, bioceramics refers to a broad category of engineered ceramics, mainly used to restore form and function of skeletal tissue structures [26]. Currently, the clinical applicability of bioceramics encompass their use as solid constructs, powders/granules, coatings, injectable formulations, and/or porous scaffolds [26].

However, bioceramics can be categorized based on their tissue response as inert or bioactive [27]. Inert bioceramics are commonly employed as femoral heads and acetabular cups for hip replacement, as well as in the production of dental implants—such as those synthesized using Zirconia [28]. However, these materials are ordinarily not utilized as scaffolds due to their inert nature and propensity to result in the development of a fibrous capsule [26]. Conversely, the capacity to establish an intimate connection between the bioceramic and host tissue is crucial for other tissue engineering and regenerative procedures, necessitating bioactive and/or bioresorbable ceramics such as HAP, tricalcium phosphate (TCP), and bioactive glass [26].

**Figure 2:**
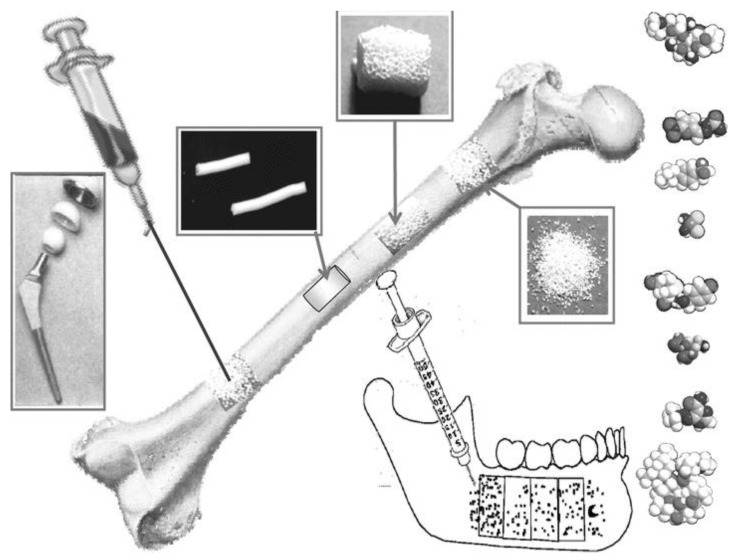
Methods by which bioceramics can administered for use in bone regeneration and repair. Reprinted with permission from Ref. [31], Copyright 2011, John Wiley and Sons.

For successful bone regeneration, these bioceramics and bioceramic devices in the form of scaffolds, coatings, injectables and particulates (Figure 2) must meet several, if not all, critical requirements, namely (i) biocompatibility, ensuring that the material does not induce an adverse immune response; (ii) porosity, essential to allow for cell infiltration, nutrient flow, and vascularization, which support tissue growth; and (iii) cell adhesion, promoting the integration of host cells with the scaffold, facilitating new tissue formation. Additionally, the bioceramic must possess appropriate mechanical properties to withstand physiological loads without compromising the healing process. The balance between bioactivity (stimulating biological responses) and bioresorbability (gradually breaking down as new tissue forms) is also crucial for the material’s success in regenerative applications. Hybrid combinations, such as co-polymers, polymer–polymer blends, or polymer–ceramic composites have been investigated to enhance these properties [29,30]. The subsequent sections focus on the primary categories of bioceramics, classified based on their microstructural characteristics. As this review covers the full range of bioceramic applications currently under research and will reference various types of bioceramic fabrications, the term ‘bioceramic devices’ will be used generically to encompass these solutions, with specific configurations such as bioceramic scaffolds clearly identified, where applicable.

## 2. Biological Background

**Figure 3:**
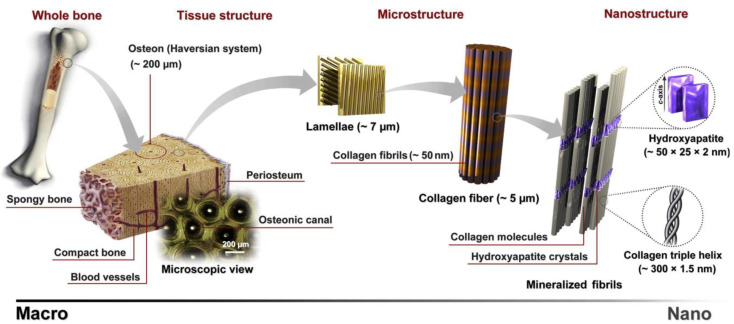
Schematic of the hierarchical structure of bone. Image reprinted with permission from Ref. [33], Copyright 2013, Elsevier Ltd.

Bone displays a hierarchical porosity (Figure 3) that spans from a single micron to several hundred microns categorized as the macrostructure, which compromises cancellous and cortical bone; the microstructure (10–500 μm), consisting of Haversian systems, osteons, and trabeculae; the sub-microstructure (1–10 μm), composed of lamellae; the nanostructure (>100 nm–1 μm), comprising fibrillar collagen and embedded mineral; and the sub-nanostructure (<100 nm), which refers to the molecular structure of constituent elements such as collagen and non-collagenous organic proteins [31,32]. When it comes to the utilization of biomaterials, it is important to consider elements that govern the development of new bone.

The successful regeneration of bone through tissue engineering relies on the replication of this natural setting by providing cells capable of differentiating into osteoblasts, initiating the release of growth factors, and employing biomaterials to facilitate cellular adhesion and differentiation. With that being said, biomaterials have undergone a distinct progression, whereby the initial (first) generation focused on substituting injured tissue, the second generation attempted to restore tissue, and the current (third) generation aims to promote tissue regeneration [31,34].

## 3. Bioceramics

**Figure 4:**
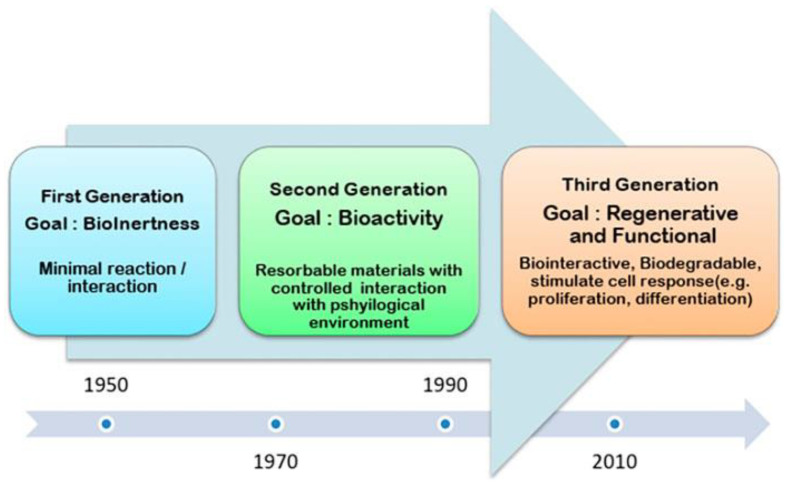
Evolution of biomaterials since the 1950s. Reprinted from [34] under the terms of the Creative Commons 4.0 CC BY license https://creativecommons.org/licenses/by/4.0/ (accessed on 29 August 2024).

Bioceramics are one of the primary forms of biomaterials currently utilized for bone regenerative procedures, with their history beings traced back to the early 1920s when calcium phosphate (CaP) materials first garnered recognition for their osteogenic capacity [35]. Prior to the 1980s, CaP had been recognized as a new material with potential for use in the repair of bony defects, assessed in the context of dentistry and orofacial reconstruction. However, by the 1980s, researchers pivoted focus to bioceramic degradability (resorption), and its dependence on density and microporosity [36]. Throughout the 1980s and 1990s, a number of bioactive glass ceramics were investigated as potential implant materials for bone reconstruction and defect repair, including Bioglass, Biocoral, microsized HAP, magnetic porous tricalcium phosphate, and mica-apatite ceramics [37,38,39,40,41]. By the early 1990s (between 1991 and 1996), glass-ceramics were successfully used in over 10,000 bone restoration procedures, although the long-term mechanical reliability of these bioceramics had not yet been fully explored [27,41]. Around the turn of the century, researchers began to emphasize not only on replacing bone defects with implant materials but on utilizing the implant to propagate regeneration of the bone owing to the intrinsic properties of the material [42]. The beginning of the 21st century marked a shift toward expanding bioactive properties of bioceramics, and advanced tissue engineering modalities (Figure 4).

Shortly after their introduction, the focus shifted to controlling the morphology and crystallinity of CaP based bioceramics, aiming to formulate an apatite with a biologically similar carbonate content and 3D lattice structure to that of native bone [43]. Research explored the osteoconduction and bone-bonding abilities of beta-tricalcium phosphate (β-TCP), a resorbable ceramic, with increased resorption and osteoconductive capacity relative to HAP [44]. With the introduction of β-TCP, research and development expanded to biphasic CaP materials composed of HAP and β-TCP of varying ratios to control resorbability, depending on the desired properties for respective in vivo applications [45,46]. Evaluation of additional additives to bioceramics such as zinc, silicon, calcium silicate, and strontium were also noted in the early 2000s [45,46]. Simultaneously, the process of 3D printing (3DP) of bioceramics took form, with rapid expansion to various applications. Following this technological innovation, the utilization of 3DP for fabricating bioceramic-based scaffolds and implants became common place, and as such, it is regularly used in the field today. In contemporary medical practice, a wide array of bioceramics is utilized in orthopedic, orthodontic, and plastic surgery procedures, to name a few, as discussed below.

### 3.1. Calcium- Phosphate-Based Bioceramics

Calcium phosphate ceramics are a versatile type of bioceramic, which have been extensively used in the field of bone tissue repair due to their surface characteristics, which promote osteoconduction [47]. Furthermore, it has been previously demonstrated that CaPs have a significant effect on stimulating bone, as well as attracting bone marrow stromal cells (BMSCs) to induce bone formation [47]. CaPs contain a calcium cation and an orthophosphate, metaphosphate, or pyrophosphate anion, sometimes with additional hydrogen or hydroxide ions [48]. The CaP family was among the first materials investigated in the class of bioceramics due to CaP, specifically HAP, being the primary mineral phase of bone - constituting 65% of its weight [2]. The initial attempt to implant CaP into fractures in pre-clinical models occurred in 1920, with significant commercialization and research of CaP bioceramics not occurring until the 1980s [35]. Today, CaP based materials are commonly used, including various types with different osteoconductivity, physical strength, and dissolution rates [48]. Nevertheless, it is important to note that different forms of CaP exhibit varying physicochemical characteristics. While the majority of these forms promote bone growth, only specific types possess the ability to stimulate bone formation [30,48]. The variations in their capacity to stimulate osteoblastic differentiation are associated with disparities in the physical and chemical characteristics [49]. Chemical features, such as surface chemistry and charge, can impact biological processes such as protein adsorption. This, in turn, can stimulate osteoblastic development through interactions between cells and the extracellular matrix [50]. In addition, surface roughness can potentially facilitate cell differentiation by impacting cell adhesion [51]. This section covers the most frequently used types of CaP ceramics, namely HAP, β-TCP, and Octacalcium phosphate.

#### 3.1.1. HAP

HAP is one of the first bioceramics selected for its chemical stability, biocompatibility, and similarity to the inorganic part of bone [15,48]. In the 1980s, early animal trials were conducted, demonstrating mixed results. To elaborate, studies by Hoogendoorn et al. and Kurosawa et al. revealed significant bone growth in surgically created defects in dog femurs, with uncomplicated healing and minimal inflammation after the initial healing phase [52,53]. HAP grafts demonstrated bone growth comparable to autologous bone grafts after 60 days. These studies concluded that HAP is an effective scaffold for bridging relatively large defects [52,53]. Conversely, Gatti et al. found no new bone growth with HAP over a 12-month period [54]. Despite early discrepancies, HAP has consistently been shown to improve bone growth in critical-sized defects, as per a systematic review by Oliveira et al. [55].

**Figure 5:**
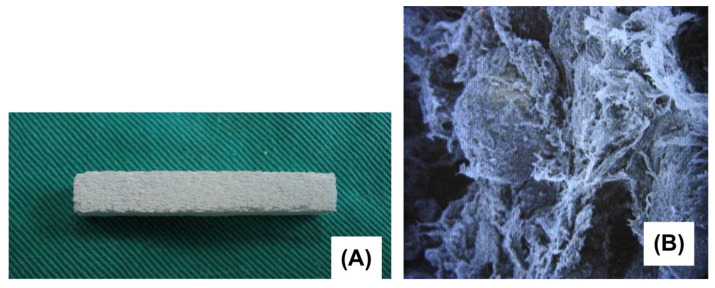
(**A**) nHAP artificial bone. (**B**) nHAP artificial bone under scanning electron microscope. Image reprinted with permission from Ref. [64] Copyright 2014, Taylor & Francis Ltd.

Significant variability exists in the type of HAP being studied, as it is available in both natural and synthetic forms with differing physical microstructures, crystal sizes, and porosities [56]. The microstructure, which includes the percentage of HAP, its shape, and its size, profoundly impacts osteoconductivity and biocompatibility. For instance, ceramics with a higher HAP percentage (around 70%) have superior biological and mechanical properties but degrade at a slower rate compared to those with lower HAP percentages (<20%) [57]. Porosity, which allows the distribution of osteogenic cells and proteins through the ceramic structure, also influences mechanical strength. However, of note, while high porosity reduces mechanical strength, low porosity decreases osteogenic capabilities [58,59]. For example, coralline HAP, with pore structures and biomechanical properties similar to human cancellous bone, is well-utilized because it provides the structural integrity needed to support joint surfaces [60]. Since their inception, coralline HAP bone graft substitutes have been shown to be clinically effective in a variety of orthopedic procedures including hindfoot surgery and distal radial fracture fixation [61,62]. Another variation, nanohydroxyapatite (nHAP) (Figure 5), is frequently combined with synthetic polymers to enhance mechanical properties and has shown safety and efficacy in procedures such as intervertebral fusions and tibial defect repairs [63,64].

Although HAP avoids the risk of donor site morbidity and patient pain due to its non-autologous nature, it is not suitable for all cases, such as socket preservation, and does not achieve the same level of bone growth as autogenous or inorganic bovine grafts in some procedures [65]. Current research has focused on surface additives/modifications of HAP bioceramic scaffolds, such as mesenchymal stem cells and platelet-rich plasma, which have demonstrated potential to enhance bone regeneration [66,67]. Although results are inconclusive, they have shown positive outcomes of bone regeneration and present a promising path forward. It is worth noting that the nanostructure of synthetic HAP does not closely resemble native HAP, leading to recent studies investigating sustainable ways to obtain HAP from natural sources through biomineralization. This complex process allows the formation of inorganic materials from living organisms, utilizing bio-waste such as eggshells [68]. For example, a review by Opris et al. found that avian eggshell-derived HAP shows similar healing characteristics to synthetic HAP and complete bone healing in an oral surgery cohort [69]. Recent studies have also investigated the incorporation of various ions into HAP to improve its osteogenic capabilities. One such study by Zhao et al. found that strontium incorporated HAP showed greater angiogenesis and bone formation relative to HAP in osteoporotic femoral rat bone defects [70].

#### 3.1.2. Beta Tricalcium Phosphate

β-TCP has gained significant attention as a potential alternative to autologous bone grafts. This is due to, in large part, its significant ability to be reabsorbed and replaced by native bone, a property lacking in some other bioceramics [71]. β-TCP is also osteoinductive, promoting osteogenesis within defect sites [72]. These attributes, combined with its similarity to the mineral phase of bone, make β-TCP a promising bone graft substitute. In a clinical trial conducted by Chung et al., β-TCP scaffolds demonstrated ~55% resorption in 12 months [73]. Other clinical studies have claimed that β-TCP possesses equivalent rates of healing, clinical success, patient improvements, and radiographic outcomes compared to autografts, with reduced therapeutic failures, pain, and complications [74]. However, the majority of studies in the literature agree that β-TCP exhibits comparable bone regeneration capacity to other synthetic implants, though not always to the gold standard, i.e., autografts [75,76].

**Figure 6:**
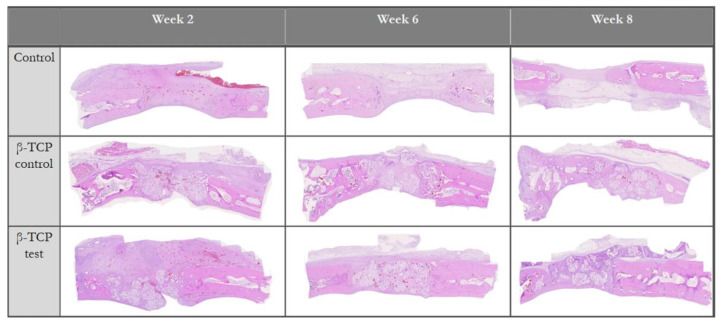
Histomicrographs of the new bone formation at the defect site treated with β-TCP (β-TCP control), or argon-glow-discharge-plasma-treated β-TCP (β-TCP test) relative to empty defects (control) at the three different time points. Reprinted from Ref. [78] under the terms of the Creative Commons 4.0 CC BY license https://creativecommons.org/licenses/by/4.0/ (accessed on 29 August 2024).

β-TCP is typically produced by solid-state reaction, thermal conversion, or direct precipitation, each method conferring specific benefits [71]. Thermal conversion, for example, produces a more homogenous material and allows for the incorporation of elemental impurities, which have the potential to increase the angiogenic, antimicrobial, or osteogenic capabilities. However, no method has explicitly been shown to improve bone grafting outcomes [71]. The surface of β-TCP ceramics can be modified to enhance the properties of these grafts, such as coating with polylactic-co-glycolic acid to improve mechanical strength and proliferation rates of surrounding bone [77]. Surface treatment with argon glow discharge plasma could also enhance bone regenerative capabilities, although this research is still in early stages (Figure 6) [78].

β-TCP can be synthesized with HAP to create a biphasic material that takes advantage of β-TCP’s rapid resorption and HAP’s mechanical strength. Preliminary clinical and preclinical studies have demonstrated promising results in bone growth, but robust pre-clinical/clinical trials are underway to fully realize its potential for bone regeneration [79,80]. To elaborate, inconsistent outcomes with β-TCP, albeit few, have been attributed to factors such as chemical composition and patient-specific variables [71]. This variability presents an opportunity for detailed investigation into the mechanisms governing its performance in bone regeneration. Understanding the influence of synthesis methods, particle size, and porosity on its biological behavior could pave the way for standardized protocols that maximize its clinical efficacy.

#### 3.1.3. Octacalcium Phosphate

Octacalcium Phosphate (OCP) is a synthetic calcium phosphate (CaP) compound with notable potential in bone regeneration. As an in vivo precursor to HAP, OCP has shown a higher affinity for fluorine ions, succinate, and DNA compared to other HAP precursors, making it a key material in bone formation [81,82]. Although discovered in the 1960s, OCP gained significant attention in the 1990s due to its rapid bone formation capabilities relative to other CaP materials [83].

**Figure 7:**
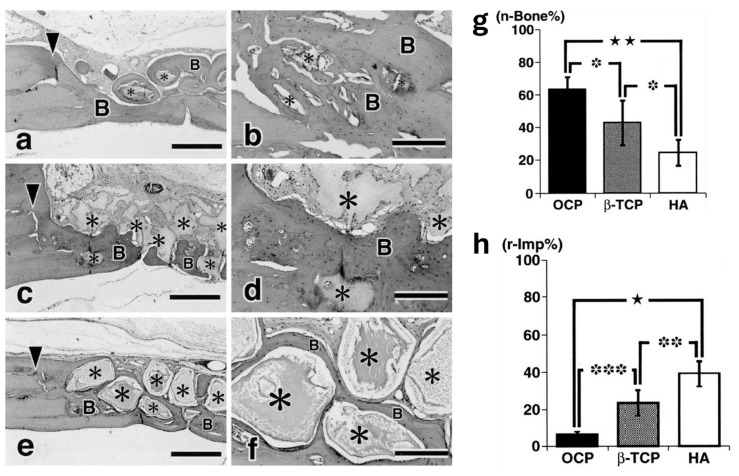
(**a**) Histological examination of the defects treated with (**a**,**b**) OCP, (**c**,**d**) β-TCP, and (**e**,**f**) HA (hydroxyapatite). (▾) depicts the margin of the defect, while (*) highlights the implanted bioceramic. Scale bars: (**a**,**c**,**e**) 300 μm; (**b**,**d**,**f**) 200 μm. Histomorphometrical examination of (**g**) newly formed bone in the defect (n-Bone%) treated with OCP, β-TCP, and HA; and (**h**) remaining implants (r-Imp%); Symbols denote statistical significance, ‘B’ = newly formed bone. Reprinted with permission from Ref. [86], Copyright 2001, John Wiley and Sons.

Studies in the literature have consistently highlighted OCP’s biocompatibility and ability to be gradually replaced by native bone. For example, Sasano et al. demonstrated that OCP induced both cartilage and bone formation within one week of implantation in long bones, with complete replacement of the cartilage by bone in two weeks [84]. Similarly, Kamakura et al. observed substantial bone growth in rat skull defects, with new bone fully replacing OCP implants within 24 weeks [85]. When compared with β-TCP and HAP, OCP demonstrated greater bone formation and faster resorption, with less remaining graft material six months after implantation (Figure 7) [86].

More recent studies have explored OCP’s ability to stimulate osteocyte differentiation. Through ionic dissolution, OCP induces earlier cellular activity around the implantation site—a feature missing in other precursor compounds like calcium-deficient HAP [87]. Leveraging this property, OCP combined with recombinant human bone morphogenetic protein-2 (rhBMP-2) has shown enhanced bone formation in rat skull defects compared to OCP alone [88]. Despite these advantages, OCP’s inherent brittleness limits its use as a standalone implant material, as it lacks the mechanical strength required to withstand significant stress [88]. In addition to its mechanical limitations, traditional fabrication processes like sintering—commonly used for other bioceramics—are incompatible with pure OCP, as they lead to thermal dehydration and a loss of molecular properties [89,90]. This limits moldability and adaptability of OCP for many applications compared to HAP.

To address these challenges, research has focused on enhancing OCP’s mechanical properties and expanding its clinical usability. For example, researchers have explored combining OCP with other materials. A notable development has been the combination of OCP with hyaluronic acid (HyA) to create a composite gel aimed at irregularly shaped bone defects. This composite has improved workability and demonstrated superior osteogenic effects compared to OCP granules alone [91]. Another key advancement is OCP–collagen composites, which have outperformed both collagen and OCP individually in promoting bone growth. Clinical trials using OCP–collagen composites for maxillofacial defects have shown promising results, with studies reporting successful bone regeneration and implant resorption without complications [92,93,94,95]. OCP coatings on non-bioactive implants represent another area of growing interest. In 2001, researchers developed a method to coat titanium implants with OCP using sequential immersion in simulated body fluids, preserving the osteogenic properties of OCP on the implant surface [96,97]. Recently, studies have explored OCP-alginate coatings, which have shown improved corrosion resistance for titanium scaffolds, potentially reducing inflammation-driven implant failure [98]. These advancements highlight OCP’s versatility, making it a promising bioceramic material for various bone regenerative applications.

### 3.2. Bioglass

Bioactive glass, also known/referred to as Bioglass, has commanded significant attention due to its distinct composition and biocompatibility. Developed by Larry Hench in the late 1960s, Bioglass is a silicate-based material enriched with calcium and phosphorus, designed to mimic the composition of native bone and stimulate new bone growth through gene activation, upregulating osteogenesis and growth factor production [99,100]. This gene activation not only promotes bone growth in fractures but also facilitates the binding of the implant to surrounding hard and soft tissues, enhancing the healing process [100]. However, as with any implant material, there is a risk of bacterial colonization. Consequently, Zhang et al. conducted a comprehensive evaluation to assess the antibacterial effects offered by various bioactive glasses. This study successfully demonstrated strong antibacterial effects against a variety of aerobic bacteria, highlighting another clinical benefit of this material [101]. Despite the versatile advantages presented by bioactive glass, there are notable limitations, including its minimal mechanical strength and decreased resistance to fracturing [100].

**Figure 8:**
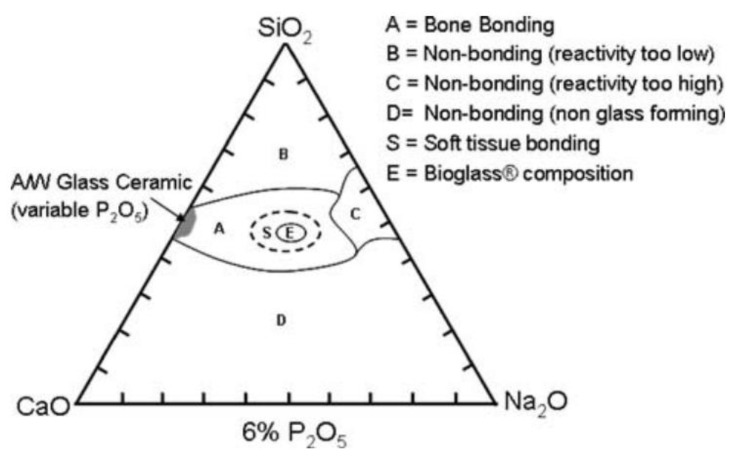
Compositional diagram of Bioglass. Reproduced with permission from Ref. [107], Copyright 2006, Springer Nature.

Over the last two decades, researchers have focused on sodium-containing bioactive glasses augmented with various other trace elements to enhance bone growth and vascularization [102]. The development of sodium-containing bioactive glasses addressed the issue of mechanical strength while maintaining bioactivity and degradability. This was exemplified by Bioglass-derived foam scaffolds, which provide transient mechanical support followed by gradual biodegradation at regulated rates [103]. Several trace elements, including copper, have been added to bioactive glass to improve bone regeneration. Copper is particularly relevant in tissue regeneration, as its addition to scaffolds has been shown to trigger endothelial cells toward angiogenesis while simultaneously providing antimicrobial properties [104,105]. The addition of borate has also demonstrated increased cell proliferation and differentiation in vitro [102]. Recent developments in this field have often focused on the utilization of a specific bioglass material, 45S5 bioactive glass (45S5 BG), with a composition of SiO_2_, 24.5% CaO, 24.5% Na_2_O, and 6% P_2_O_5_, with various other composite-based enhancements (Figure 8) [106,107].

A study by Korukonda et al. assessed a novel bioglass composed of silver and gadolinium, demonstrating a 10% increase in osteogenicity relative to standard bioglass, while simultaneously maintaining antibacterial activity [108]. These characteristics of the composite can be attributed to the silver content exhibiting antibacterial activity, enhancing osteogenic cell growth without posing an increased toxicity risk to target cells. Alternatively, gadolinium regulates cell behavior and bone regeneration by indirectly expediting osteogenic cell differentiation and scavenging reactive oxygen species. [108]. This finding is significant for clinical application as it suggests that further improvements in bone regeneration can be achieved while reducing negative effects such as tissue damage, inflammation, and potential infection [109]. Another recent study by De Mori et al. combined 45S5 BG with polycaprolactone (PCL)-based scaffolds to increase the load-bearing ability of the material for use in large bone defects [110]. PCL is a thermoplastic known for its strength and ability to integrate into tissues, which is beneficial when combined with bioglass for repairing defects and promoting bone regeneration in areas of large bone loss [111]. This study demonstrated that the PCL-bioglass combination enhanced bone formation in the center of the defect in load bearing sites [110]. Such findings hold clinical potential, as a primary limitation of bioglasses is their inherent lack mechanical strength, limiting their application in load-bearing sites. However, this investigation suggests a potential breakthrough in utilizing these materials across a wide range of locations for bony defect repair [100]. A recent study has assessed the addition of luminescent material into bioglasses to create nanoparticles for biological labelling and drug delivery applications [112]. This approach offers various benefits including providing information about the biodegradation of bone implant materials and represents an intriguing area for future study [113]. As demonstrated, bioglasses present a diverse spectrum of benefits, especially when combined with complementary materials and molecules to form composites that capitalize on their collective physicochemical and biological benefits.

### 3.3. Composites and Polymers

One potential way of improving the physical, chemical, and biological response of bioceramics for use in bone tissue engineering and regeneration has been indicated to be through their combination with polymers [114]. As stated previously, polymers are organic materials composed of long chains of atoms joined by covalent bonds, and are large molecules found in nature and in the human body. However, their use as a biomaterial is limited mainly due to rapid and uncontrolled degradation, as well as poor mechanical strength [115]. Nevertheless, they can be combined with CaP ceramics, such as HAP and β-TCP, to enhance the biocompatibility and cell adhesion characteristics [116]. Composites, any combination of two or more materials, are created with the intention of combining favorable properties from each of the constituent materials. They can be formed as cements, pastes, coatings, and hydrogels, each with its own uses and disadvantages. For example, bioceramic composite pastes are used to make highly porous scaffolds and are advantageous for 3D printing, while hydrogels are networks of hydrophilic polymers, which allow for customization of their stiffness and viscoelasticity [31,117]. Bone cement, as the name suggests, is significant due to its ability to self-harden upon injection and has been approved for hip and knee prosthetic fixation since the 1970s. However, they are largely neither osteogenic nor resorbable [48].

Polysaccharides are one of the most common polymer types in bone regenerative applications owing to their natural participation in hard tissue formation, mineralization, and collagen fibrillogenesis. Natural polysaccharides are limited in their application due to their rapid and uncontrolled degradation, mediocre mechanical strength, and variability of molecular weights. However, these limitations have been addressed through their incorporation with other material systems [115,116,118]. For example, chitin and chitosan, cationic polysaccharide substances that form the exoskeleton of arthropods and outer skeleton of shellfish, are two of the most abundant biopolymers [24]. They are examples of polymers that can be combined with bioceramics to achieve a more ideal implantable material. For instance, Amirthalingam et al. combined bioglass nanoparticles with FGF-18, a fibroblast growth factor that can enhance osteogenic differentiation, to create a chitin-poly(lactic-co-glycolic) acid hydrogel. This composite showed new bone formation greater than commercial bioceramics and a higher synergistic effect [119]. These materials can also be used to enhance the properties of scaffolds, as shown in a study by Hu et al., where a novel crosslinking technique was used to create a chitosan–vanillin–bioglass scaffold that was antibacterial, osteoconductive, and highly porous [120]. This scaffold promoted osteoblastic differentiation and mineralization in addition to its enhanced mechanical properties, making it promising for bone tissue engineering [120]. Scaffolds of this composite can be further specialized to fit specific needs be the addition of other particles, such as akermanite particles which may increase cell proliferation and migration of epidermal cells [121].

Draghici et al., on the other hand, took advantage of the porosity and purity of cellulose, the osteointegration and osteoconduction of CaP, and the piezoelectric and ferroelectric properties of barium titanate to create a stable bioceramic capable of delivering electrical stimulation to the microenvironment, potentially further supporting cell viability [122]. More commonly though, cellulose is combined with HAP, particularly in a cellulose nanocrystal–HA nanohybrid. This composite enhanced mechanical strength, thermal stability, and antibacterial activity in vitro, and has the potential to further enhance cell viability when combined with materials like sodium alginate and polyvinyl alcohol [116,123]. Anionic polysaccharides, most notably alginate-based, are another group of polymers utilized for composite bioceramics. Alginate is a water-soluble compound found in seaweed and bacteria that is known for its flexibility, thermal stability, and mechanical strength. When combined with α-tricalcium phosphate microparticles, the composite was capable of controlled release of bovine serum albumin and dimethyloxaloylglycine, while maintaining biocompatibility [124].

As the field of bioceramic composites continually evolves, the potential for creating the ideal bone graft substitute continues to grow. The integration of polymers with bioceramics not only improves the mechanical and biological properties of the materials but also opens new avenues for incorporating cells and signaling molecules, further advancing the capabilities for bone regeneration (summarized in Table 1). Through continued research and development, these composite materials hold the promise of revolutionizing the approaches to bone healing, offering more effective and tailored solutions for patients.

## 4. Methods of Bioceramic Device Fabrication

**Figure 9:**
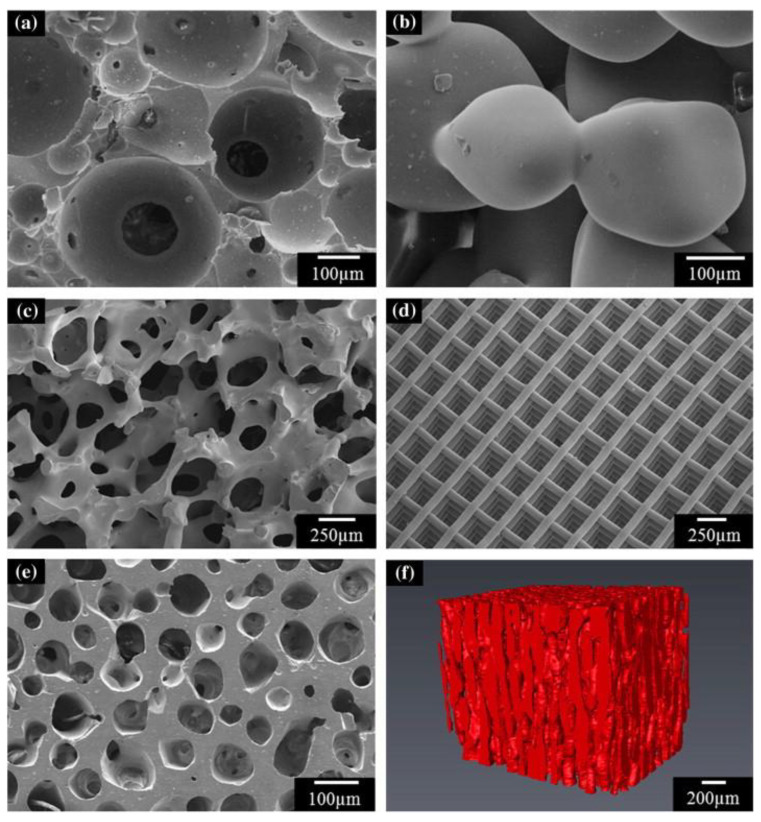
Microstructures of bioactive glass scaffolds synthesized by (**a**) sol-gel; (**b**) sintering; (**c**) polymer foam replication; (**d**) Robocasting (3DP); and (**e**) unidirectional freezing. (**f**) Micro-computed tomography image scaffolds shown in (**e**). Reprinted with permission from [125], Copyright 2011, with permission from Elsevier.

Various processing methods have been extensively explored in the literature for the synthesis of bioceramic constructs (scaffolds) for tissue engineering (summarized in Table 2). These techniques encompass methods that have been developed spontaneously or frequently modified from other techniques, offering a way to tailor the three-dimensional arrangement of constructs for tissue engineering, which can affect bioceramic device behavior in in vitro and in vivo settings (Figure 9). Therefore, it is imperative to select the most suitable approach that precisely fulfills the requirements of the tissue requiring reconstruction and regeneration. Furthermore, each manufacturing method varies in terms of production and post-processing costs rendering certain methods appealing for mass production, while others are better suited for customized patient- and anatomic location-specific treatment modalities.

### 4.1. Sintering

Traditional methods of solid implant production, primarily revolving around powder processing and sintering, have laid the foundational framework for bioceramic fabrication. Calcium phosphate ceramics have been produced by a solid-state reaction incorporating various powder constituents, followed by shaping and sintering at high temperatures, typically between 600 °C–1550 °C [126]. This process of heating powders to create a solid ceramic structure can be viewed as a balance between densification and grain growth [127,128]. During this final sintering stage, the chemical composition, particle shape, and size may be altered, underscoring its importance in determining the final properties of the product [127].

Some of the advantages of this method include its single-step procedure, rapid turnaround, and the production of high-purity products, typically at a low cost [33,113]. The cost of sintering in the production of biomaterials varies, depending on factors such as equipment required, temperature, and energy consumption. Due to high temperatures, sintering is an energy-intensive process that can quickly contribute to costs on both laboratory and industrial scales [129]. The materials produced by high temperature sintering techniques are generally regarded as biocompatible and elicit minimal immunogenic response in the body [128]. However, some studies, such as Doherty et al., have noted a lack of biocompatibility in certain bioceramic materials [130]. Porosity is a critical element in producing bioceramic implants, as it is essential in promoting favorable processes like cell infiltration and vascularization [131]. This pivotal role stems from the bone’s ability to infiltrate these constructs, where pores of 100 µm are necessary for containing intracellular and extracellular tissue components, while pores of 200 µm are essential for facilitating osteoconduction [132,133]. The porosity of a bioceramic can also be manipulated to a degree via sintering, with the addition of organic materials which are burned out of the ceramic, resulting in macropores, termed organic phase burn-out. Conversely, sintering at lower temperatures can contribute to microporosity [134]. However, the intrinsic limitations of these techniques, such as the challenge in precisely controlling porosity and the risk of structural weaknesses, have driven the development of advanced methodologies [33,113]

### 4.2. Sol-Gel Technique

Within the scope of bioceramic scaffold production, the sol-gel technique represents a paradigm shift toward synthesizing bioceramics with enhanced functional properties. Initiated in the 1990s, this wet-chemical technique has facilitated the creation of bioceramics that are not only homogenous at the molecular level but also customizable in terms of their chemical composition and microstructure [135]. This method of production begins with small molecules of precursor monomers mixed into a solution that then undergo hydrolysis and condensation into the polymers of the gel [135,136]. Prior to the condensation into a three-dimensional structure, the gel can be poured into a mold, allowing for precise shape of the gel product after drying.

In addition to homogeneity and customizability, the sol-gel method yields numerous benefits that researchers have leveraged, including improved purity, lower temperature requirements, and nanoporosity [136,137]. Low-temperature reactions are essential for reducing costs and environmental impact compared to traditional high-temperature combustion methods for bioceramic scaffold production. This advantage contributes to the scalability of production for large-scale commercial applications [138]. Meanwhile, the nanoporosity results in an increased bioactivity and mimics the native structure and physiological environment of bone tissue [125]. Cholewa-Kowalska et al. assessed two gel-derived bioglass materials as modifiers of traditional HAP ceramics, showing that the addition of both high and low amounts of silicon improved cell growth, cell viability, and cellular activity [139]. Adding these gel-derived bioglasses to HAP thereby combines their properties, enhancing the bioactivity and microstructure of these bioceramics.

In the past five years, studies involving sol-gel derived biomaterials have become ubiquitous as researchers have come to recognize their benefits as multifunctional nanocarriers in a wide range of applications. For example, Naruphontjirakul et al. investigated the application of microemulsion-assisted sol-gel-derived bioactive glass nanoparticles incorporating strontium and zinc for potential use in bone tissue engineering [140]. The microemulsion process facilitates the synthesis of size-controlled nanoparticles, enhancing the precision in creating bioceramics with the desired composition, size, and shape [141,142]. It was discovered that adding strontium increased osteoblast activity while inhibiting osteoclast activity, leading to increased bone formation, while zinc enhanced osteoblast activity and bolstered antimicrobial properties. This exhibited multiple functionalities, including bone regeneration, intracellular therapeutic ion delivery, and inhibition of bacterial growth, suggesting its usefulness in clinical application [140]. Another study by Viegas et al. determined that the sol-gel method of producing a polydimethylsiloxane–silica (PDMS–SiO_2_) system showed potential for incorporating resveratrol for enhanced osteogenic, osteoinductive, and antitumoral qualities, acting as a biomaterials for delivery systems [143,144]. This sol-gel derived material provided increased solubility and enhanced released of resveratrol, revealing its potential use in treating osteosarcoma [144].

Despite its significant advantages, the sol-gel process is associated with its respective challenges. The method’s susceptibility to cracking during the drying phase, resulting from capillary stresses as solvents are removed, requires careful management to preserve the scaffold’s integrity [145]. Additionally, there are limitations in producing exclusively glassy products, as studies have shown it difficult to eliminate crystalline phase inclusions entirely [136,146]. These crystalline phases of HAP can be beneficial in promoting increased mechanical strength and bioactivity; however, excessive inclusions within the gels can reverse these benefits, rendering the material more fragile and impairing bone bonding abilities due to this heterogeneity [136]. As research in scaffold production continues to advance, the sol-gel process stands out for its potential to revolutionize the development of bioceramics for bone regeneration. By addressing its limitations and exploring new formulations, this technique can further contribute to creating bioceramic scaffolds that offer improved outcomes for patients requiring bone repair and regeneration, highlighting ongoing innovation within the field.

### 4.3. 3D Printing

3D printing (3DP) is a rapid prototyping technique that allows the creation of customized patient- and anatomic-location-specific scaffolds or tissue engineering devices and has revolutionized the field of bioceramics in bone regeneration [147]. In recent years, the rapid advancement of 3DP technology has transformed the production of bioceramics, enabling the creation of complex scaffolds with unprecedented accuracy, fidelity, detail, and chemical composition [148,149,150,151]. Earlier modalities of 3DP were characterized by their high initial costs, high energy requirements, and low reliability. However, over the past few decades, 3DP has seen rapid progress owing to its versatility, rendering it a highly efficient and reliable method, ideal for a patient-centric approach over population-centric creation of scaffolds and tissue engineering devices [152].

The primary modalities of 3DP bioceramics include extrusion-, droplet-, or laser-based techniques, each with its own set of advantages and disadvantages. Extrusion-based printing utilizes a dispensing system to precisely deposit feedstock onto a substrate. It is an affordable 3DP technique and has greater deposition volumes and printing speeds. However, it has been reported to typically enable the production of lower resolution end-products [153]. Droplet-based printing, or inkjet printing, utilizes gravity, atmospheric pressure, and the intrinsic rheological behavior of the feedstock solution to generate droplets, which are then ejected onto a substrate with high reproducibility. However, it has been noted that droplets may gel in the air prematurely, causing unintended defects in the printed parts [154]. Finally, laser-based bioprinting uses laser direct-write technology, which is advantageous due to the absence of direct contact between the bioink, and feedstock dispenser used in the extrusion- and droplet-based technologies. This has shown to result in higher cell viability (pertaining to the use of stem cells in conjunction with the feedstock material). However, the effects of laser exposure on cells are not yet fully understood [155].

However, unlike both conventional and sol-gel methods, 3DP does not require a mold for bioceramic fabrication, and can utilize medical imaging to directly create patient-specific scaffolds [147]. Despite these advancements, 3DP of biomaterials remains a challenge. Ceramic melting points are exceptionally high, and their mechanical properties are not ideal for currently available 3DP technology [156]. For example, 3DP of bioceramics often requires the use of binders (to enable to adhesion of ceramic powders); however, this has yet to be optimized for osteoconductivity and biocompatibility in clinical applications. Liquid binder solutions are often acidic, which decreases biocompatibility [156]. One study by Inzana et al. attempted to optimize the binder solution by tailoring the phosphoric acid-based binder to 8.75 wt% and adding Tween 80, a non-cytotoxic surfactant, and collagen to the composite material [157]. This was found to be osteoconductive, with optimized material parameters in critically sized femoral defects after nine weeks, offering a potential avenue for overcoming the challenges of 3DP in the future [157].

### 4.4. Cost of Fabrication Methods

Despite the considerable advancements that have been made in the synthesis of biomaterials, including methods like sintering, sol-gel processing, and 3D printing, there is a notable gap in the literature regarding the specific costs associated with these techniques. Existing research instead has prioritized optimizing the material properties, bioactivity, and mechanical strength, rather than providing detailed economic analyses of these processes. However, understanding the financial implications of each method is critical for determining their scalability and practical application, particularly as the field progresses toward larger-scale manufacturing and clinical adoption. As such, the economic feasibility of each method, alongside its technical efficacy, should be a focus for future studies. Factors of production such as equipment costs, energy consumption, and accessibility of materials may contribute to viability of these technologies in clinical practice. Addressing these gaps will aid in the development of more cost-effective production strategies for widespread use.

## 5. Improving Bioactivity and Capacity for Clinical Applications

As previously demonstrated, a wide range of biomaterials have been studied preclinically to assess osteogenesis, osteoinduction, and osteoconduction for bone regeneration; however, few have moved onto clinical studies [29,158,159]. The preclinical evaluation of selected biomaterials and the cell product is a mandatory step before starting a clinical trial, according to regulatory agencies [29]. However, as bone tissue engineering progresses/transitions toward clinical application, it becomes crucial to demonstrate the therapeutic efficacy of these innovative tissue engineering devices [29]. In response to the growing demands of patients, as well as the significant reduction in their age range, surgeons have been adopting new surgical techniques and designs in bone reconstructive procedures [160]. In the context of surgeries focused on bone tissue reconstruction, preventing potential complications is closely associated with the design of these devices [161]. As such, functionalization involves modifying the material to enhance its interaction with native biological tissues, promote desired cellular responses, and facilitate the regeneration process. This can be achieved through a variety of methods that tailor the device’s physical, chemical, and biological properties. The primary goal is to create an environment that closely mimics the natural extracellular matrix, thereby improving the device’s performance in clinical applications. Key methods employed to functionalize tissue engineering devices (such as prostheses and scaffolds), include the incorporation of bioactive molecules, the seeding of stem cells to enhance tissue regeneration, and the influence of microarchitectural design and surface topography on cellular behavior to not only support but actively participate in the healing and regeneration process [162,163].

### 5.1. Bioactive Coatings

**Figure 10:**
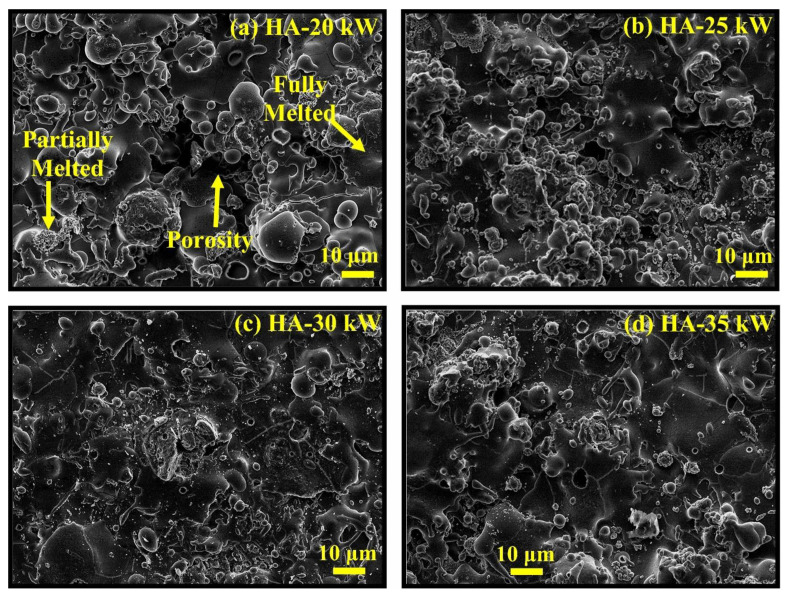
Scanning electron micrographs of the surface microstructure of plasma-sprayed HA (hydroxyapatite) coatings at different plasma spray settings. Reprinted with permission from Ref. [164], Copyright 2020, with permission from Springer Nature.

Bioactive materials are utilized for bone regenerative procedures in large part due to their ability to enhance osteointegration. Bioactive calcium phosphates and silica-based glasses have exceptional characteristics as bone grafts in non-load-bearing applications, prioritizing bone regeneration kinetics. Regarding their use as metal coatings, one commonly utilized method involves applying calcium phosphates over titanium alloys, whereby the underlying bulk metal provides structural reinforcement while the ceramic surface coating exhibits bioactive properties. Furthermore, the application of bioceramic coatings, as previously mentioned, inhibits corrosion of the metal substrate. Bioceramic coatings are typically carried out using plasma spray owing their fast deposition rate and low cost (Figure 10) [164]. Nevertheless, other methods include physical vapor deposition, chemical vapor deposition, magnetron sputtering, electrophoretic deposition, pulsed laser deposition, and dip coating, to name a few [165].

### 5.2. Drug Delivery Vehicles

In recent years, the intersection of bioceramics and drug delivery has led to notable progress in the field of bone regeneration. Drug-loaded bioceramics offer the potential of serving as biocompatible, resorbable scaffolds with the added benefit of controlled, sustained delivery of therapeutic agents and bioactive signaling molecules to bony defect sites. This approach offers benefits such as increased expression of tissue-inductive factors to enhance the differentiation of progenitor cells into osteoblasts or blocking the expression of factors inhibiting autologous bone regeneration [166]. Collectively, the incorporation of drug delivery systems into bioceramic scaffolds has shown enhanced healing and increased bone regeneration, as well as various antibacterial and anti-inflammatory benefits [166,167].

Numerous studies have assessed combinations of bioceramic backbones and therapeutic agents loaded onto them. For example, bioglass has been investigated as a potential drug delivery vehicle, showing success in a study that assessed vancomycin delivery for treating osteomyelitis [168]. Additionally, Butler et al. explored the role of various individual steroids, including testosterone, dihydrotestosterone, and androstenedione, delivered by β-TCP bioceramics on the fibrous tissue forming around the site of implantation [169]. It was observed that each hormone loaded onto the bioceramic significantly influenced the thickness and cellular composition of the fibrous capsule, especially androstenedione [169]. Furthermore, hormone delivery significantly decreased cytokines expression at the defect site, indicating potential for modulation of the immune response with therapeutic agents to improve healing outcomes surrounding bone implants [170]. While beneficial for drug delivery, bioceramics pose limitations, such as burst drug release, where a large bolus of the drug is released immediately after implantation, posing potential pharmacological concerns [171,172]. HAP–alginate composites have been shown in recent years to be useful in providing sustained drug delivery, overcoming the issue of burst drug release and facilitating a prolonged gradual release of therapeutic agents over time [171,173].

In more advanced use-cases, nanotechnology’s role in drug-delivering bioceramic models is particularly advantageous due to properties such as an increased surface area-to-volume ratios [174]. Nanobioceramics offer a unique approach to bone regeneration due to their particle size between 10–100 nm, which positively impact their biological response and capabilities [175]. Bioceramic nanocomposites have been found to be superior in achieving gradual controlled release of various medications for treating bone defects [176,177,178]. Nanoscale modifications to bioceramics can create porous networks serving as reservoirs for therapeutic agents, such as antibiotics, allowing for controlled release directly at the injury site [179]. Studies have shown promising results, where adding quercetin to bioceramic microspheres can induce osteogenesis and angiogenesis in vivo, while also inhibiting osteoclastogenesis in osteoporotic conditions, offering optimistic avenues for therapeutic intervention in osteoporotic bone regeneration [180]. In osteosarcoma treatment, research on nanotherapeutics in bioceramic scaffolds have aimed to repair critical defects left after bone tumor resection, repress tumor growth, and support healthy bone regeneration [174]. Overall, the addition of therapeutic agents to bioceramic scaffolding, particularly nanomaterials, has shown greater healing and new bone formation with antibacterial and anti-inflammatory activity compared to control scaffolds without drug-loaded systems both in vivo and in vitro [167].

Despite their promise, nanomaterials have been indicated to potentially result in systemic damage through their deposition in organs, by penetration of cell membranes, and/or through the triggering of an adverse response [181]. Though this has not been shown specifically through the use of nanobioceramics, Feng et al. proposed potential pathways of central nervous system toxicity due to the ability of nanomaterials to cross the blood–brain barrier, to trigger cell apoptosis, and to exploit endocytosis [181].

**Figure 11:**
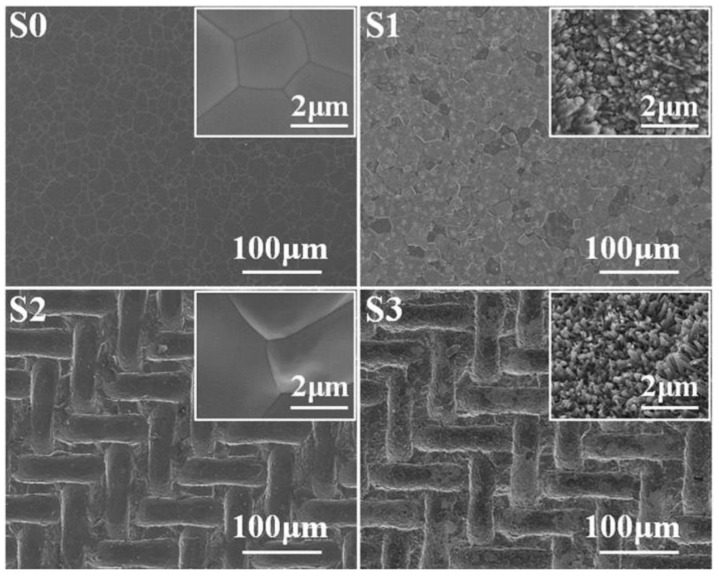
Scanning electron micrographs and high magnification images (corresponding inserts) of nanotopography including (**S0**) smooth; (**S1**) nanorod; (**S2**) micropatterned; and (**S3**) micropattern/nanorod surface structures. Reprinted from [182] Copyright 2018, with permission from Elsevier.

The goal of modifying the nanotopography is to mimic the nanostructure of natural bone, potentially improving the regulation of stem cell differentiation, tissue regeneration, and cell adhesion (Figure 11) [182]. Specifically, micro- and nano-HAP topographies have been shown to better facilitate adhesion, spreading, and osteogenic differentiation of bone marrow stromal cells, which may translate to improved in vivo osteoinductivity [183]. The sintering technique is critical in the composition of nanomaterials due to the difficulties associated with maintaining particle size and microstructural design. However, despite assisted sintering techniques being considered advantageous, they do not seem to correlate with increased HAP mechanical reliability compared to traditional sintering methods [127].

### 5.3. Immunomodulatory Agents

The implantation of bioceramics may cause an immune reaction, which includes a mounted response from various cells such as neutrophils, macrophages, and multinucleated giant cells, and could impact the osteogenic ability of the bioceramic and/or hamper the healing capacity of the defect [184]. For example, osteal macrophages may play a role in typical bone healing and bone regeneration, although this area requires further research [185]. Some research groups have been able to manipulate this immune environment to facilitate an ideal microenvironment. An example is the incorporation of ions, such as magnesium, lithium, and strontium, which have been shown to suppress inflammatory cytokine secretion and promote tissues regeneration, including peripheral nerves [186,187]. Additionally, other bioactive molecules, such as HyA and fibrin, have been shown to recruit non-inflammatory cytokines, which promote bone healing when added to bioceramics [188,189].

Of note, negative surface charges and hydrophobic surfaces have been shown to increase interleukin-10 (IL-10), an anti-inflammatory cytokine, and decrease interleukin-8 (IL-8), an inflammatory cytokine [190]. This suggests that surface treatment with polymers or production of bioceramics with hydrophobic properties could perhaps also facilitate improved bone healing through mitigation of the immune response, though this association has not yet been well-established. Similarly, rough bioceramic surfaces may recruit more M2 macrophages, which secrete IL-10, while smoother surfaced implants may tend to recruit M1 macrophages, which secrete interleukin-1 (IL-1), a pro-inflammatory cytokine [188]. Additionally, larger pores may be associated with a lower number of inflammatory cells compared to scaffolds with smaller pores, and this difference has been shown to increase over a 28-day time period in a histological analysis of critical mandibular rat defects implanted with composite bioceramics with varying pore sizes [191].

### 5.4. Cells and Signaling Molecules

#### 5.4.1. Bone Marrow Stem Cells

Since the turn of the century, tissue engineering approaches for bone have shifted towards the integration of cultured, living stem cells into bioceramic scaffolds with the goal of targeted delivery of these integral cells to the site of bone defects [192]. Mesenchymal stem cells (MSCs) are progenitor cells with the ability to differentiate into various cell lineages depending on inductive cues. They also serve to secrete growth factors that promote cell proliferation, tissue survival, and angiogenesis [193]. When bone marrow MSCs are loaded onto a bioceramic scaffold and implanted, they form a highly vascularized primary bone tissue [192]. Due to the multilineage differentiation capabilities arising from these cells, they can be manipulated to differentiate into various tissue types, including bone and cartilage [194]. Previously, materials such as collagen coated polymer beads and polymer scaffolds had been used with less success [194,195,196]. However, bioceramics have been successfully assessed in this context as microcarriers of bone marrow stromal cells and osteosarcoma cells, both promoting the formation of native bone when implanted. This avenue of study is vast with much remaining to investigate, including clinical applications in patient populations.

A recent study by Dong et al. investigated the implementation of silicon ions on bioceramic scaffolds seeded with MSCs to combat the negative osteogenic effects of a high-glucose environment [197]. It has been recognized that hyperglycemia impairs bone quality, and that high glucose levels cause inhibition of osteogenic differentiation of bone marrow stem cells, resulting in diminished bone regeneration capabilities [198]. This study proposed that silicate bioceramics stimulate bone regeneration and that the release of silicon ions from these materials could enhance osteogenesis and bone marrow stem cells to a degree, compensating for the negative impact of high levels of glucose [198]. It was shown that in the setting of high glucose environment, silicate bioceramics were able to promote the proliferation and differentiation of stem cells, overcoming the inhibitory effect of elevated glucose concentrations [198]. This is critical in clinical applications, as it allows for the expansion of use of bioceramics in bone regeneration of diabetic patients who otherwise may have less autologous regenerative potential than non-diabetic counterparts in addition to an increased risk for osteoporosis and fracture [197,199].

Other studies have also shown the potential for the culturing and utilization of specific MSC cell populations for bone regeneration. Zhang et al. used periodontal ligament stem cells (PDLSCs) in contrast to bone marrow stem cells (BMSCs) as the seeded cell population in testing the performance of a collagen-based matrix to repair large bone defects [200]. The results demonstrated outcomes similar to those expected from BMSC-seeded implants, suggesting a possible alternative to BMSCs in the future [200]. This opens the possibility of utilizing tailored MSC populations for different use cases and implant types, potentially reducing the necessity of highly invasive procedures like bone marrow harvesting.

#### 5.4.2. Vascular Endothelial Growth Factor (VEGF)

Angiogenesis is another critical component of regenerative medicine, as it allows blood flow to be supplied to newly regenerated tissues, facilitating the integration of bioceramic implants [201]. Vascular endothelial growth factor (VEGF) remains one of the most potent molecules for induction of vasculature formation in regenerative medicine and acts as a fundamental rate-limiting step in angiogenesis [202,203]. While VEGF in bioceramics is a potentially untapped area of study, there remains much debate about its ability to incite a response in regenerative bone tissue. Huang et al. found that VEGF in combination with osteogenic factor bone morphogenic protein 4 (BMP-4) and MSCs on implanted scaffolding in mice resulted in improved bone formation and angiogenesis [204]. Meanwhile, another study found that the local application of VEGF to regenerating bone did not elicit a response in regenerating bone, while native bone responded to its application with increased angiogenesis [205]. Due to these challenges with inducing angiogenesis via the addition of VEGF alone, one study explored phage nanofibers as a potential method to induce blood vessel formation by exploiting cell–cell and cell–matrix adhesive interactions. They found that these phages were successful in increasing vascularized bone tissue, but even more so with the addition of VEGF, solidifying the significance of this molecule in promoting vascularization in bone regeneration [206]. 

## 6. Future Directions

### 6.1. Silicate Bioceramics

One area of future interest and development is the introduction of silicate bioceramics (SB). Similar to OCP, SBs demonstrate exceptional bioactive and physiochemical properties [207]. Also similar to OCP in its early stages of research, there is still debate surrounding ideal manufacturing processes and the mechanistic understanding of its bioactivity [207]. The variety of composites under the umbrella of SBs also lends itself to the lack of definite direction surrounding this novel bioceramic [208]. Current clinical uses are mostly within dentistry, where calcium-silicate-based cements (CSCs) and calcium-silicate-based sealers (CSSs) have found various applications [208]. Many of the properties that made OCP desirable also make CSCs and CSSs desirable, including biocompatibility and bioactivity. However, these compounds have additional properties that make them particularly suitable for dental applications, such as their sealing ability and the capability of setting in humid conditions [208]. Many variants of SBs are being explored to discover new properties, including baghdadite, a calcium-silicate biomaterial with trace inclusions of zirconia within its lattice structure [209].

It is one of several possible offshoots of SBs that can be produced, but a more promising one, due to its adaptability to several methods for scaffold production, from sintering to 3D printing to composite formation [210]. Some studies have even looked at producing multilayered, sintered SB and non-SB bioceramic implants to better control the implants evolution post-implantation [211]. For example, researchers created an implant with a β-TCP outer shell and a calcium-silicate core of slightly varying composition to test the different in vivo behaviors of these variants [211]. Other studies have also explored the applications of SB beyond hard tissue use-cases, including chemotherapy drug delivery and soft tissue regeneration [212,213]. While the applications for SB within the larger bioceramic use is still limited, its trajectory for research and adoption for clinical use is promising and could play a larger role as bioceramics continue to gain wider acceptance.

### 6.2. Antioxidants

Reactive oxygen specific (ROS) scavenging molecules, or antioxidants, may be loaded onto nanobioceramic implants to decrease harmful effects like cellular senescence, fibrotic scarring, and inflammation from ROS damage [214,215]. Na et al. investigated alpha-tocopherol loaded onto capsules of ferrocene, a unique organometallic compound with capabilities of redox activity, ROS scavenging, and ability to convert between two states after oxidation, which would result in drug release upon transitioning from hydrophobic to hydrophilic [216,217]. This combination of alpha-tocopherol on the ferrocene capsules demonstrated significantly higher antioxidant properties and improved wound healing [217]. Another recent study found that manganese containing bioceramics inhibit osteoclastogenesis and promoted bone regeneration by similar ROS scavenging properties [218]. This further underscores antioxidants and scavenging ROS as a potential avenue of research in bone-regenerating bioceramics.

## 7. Conclusions

Since their inception, bioceramics have evolved to include a vast variety of materials, production techniques, and surface treatments, offering many viable alternatives to autologous bone grafts. This paper comprehensively reviewed the applications of these variations on bioceramic production and composition while analyzing the various strengths and weaknesses of each method and material. The breadth and depth of the literature analyzed highlights the importance and the interest in creating an ideal bioceramic for bone regenerative applications. There are many promising directions forward, including the investigation of new cells and signaling molecules, silicate bioceramics, more efficient 3DP, new composites, and new surface treatments. However, this review does highlight that an ideal bioceramic has not yet been developed, and despite the large volume of research on the topic, current bioceramics do not yet fully outpace autologous bone grafts. However, with continued research and development, we can expect bioceramics to reshape the future of bone regenerative procedures, improving patient outcomes and revolutionizing the field.

## Figures and Tables

**Table 1:** Key summary of the materials discussed.

Material/Material Type	Details	Key Advantages	Key Limitations
Hydroxyapatite (HAP)	Biocompatible, chemically stable, and similar to the chemical composition of bone.	Excellent biocompatibility and osteoconductivity, stable in physiological environments.	Slow resorption, not ideal for rapid bone remodeling.
Beta-Tricalcium Phosphate (β-TCP)	Good safety and resorption profiles.	Resorbable, ideal for rapid bone healing and regeneration.	Lower mechanical strength, resorbs faster than the bone healing rate.
Octacalcium Phosphate (OCP)	Precursor to HAP, used for bone regeneration.	Promotes faster bone formation as a precursor to HAP.	Poor mechanical strength, brittle when used alone.
Bioactive Glass	Silicate-based material with calcium and phosphorus, designed to stimulate bone growth through gene activation.	High bioactivity, promotes bone bonding and regeneration.	Brittle, limited mechanical strength.
Composites and Polymers	Combinations of materials, often including bioceramics and synthetic or natural polymers.	Can combine mechanical strength with bioactivity, tailored to specific applications.	Requires careful formulation to balance mechanical properties with biocompatibility.

**Table 2:** Summary of key points related to the commonly used fabrication methods.

Fabrication Method	Details	Cost	Complexity	Development Cycle
Sintering	Traditional powder processing technique involving heating to create a solid/densified structure.	Low	Moderate	Long (time-intensive, due to the high temperatures required for densification).
Sol-Gel Method	Produces bioactive glasses and CaP materials, offering a moderate level of control over porosity and mechanical properties.	Moderate	High (precision needed)	Medium (offers flexibility but requires precise temperature control).
3D Printing/Robocasting	Advanced method allowing for custom, complex scaffolds with engineered pore architectures, and improved mechanical properties.	High	High (requires technical expertise)	Short (relatively fast process, but post-processing can be time-consuming depending upon the modality and materials used).

## Data Availability

No new data were created or analyzed in this study. Data sharing is not applicable to this article.
